# Normal Anatomy and Variants of Renal Vasculature with Multidetector Computed Tomographyin a Tertiary Care Hospital: A Descriptive Cross-sectional Study

**DOI:** 10.31729/jnma.5615

**Published:** 2020-11-30

**Authors:** Pradeep Raj Regmi, Isha Amatya, Prakash Kayastha, Sharma Poudel, Sundar Suwal, Ram Kumar Ghimire

**Affiliations:** 1 Department of Radiology and Imaging, HAMS Hospital Private Limited, Kathmandu, Nepal; 2 Department of Radiology and Imaging, Kathmandu Medical College and Teaching Hospital, Sinamangal, Kathmandu, Nepal; 3 Department of Radiology and Imaging, Tribhuvan University Teaching Hospital, Maharajgunj, Kathmandu, Nepal; 4 Department of Radiology and Imaging, Nepal Mediciti Hospital, Karyabinayak, Lalitpur, Nepal

**Keywords:** *artery*, *nephrectomy*, *renal*, *variations*, *vein*

## Abstract

**Introduction:**

Preoperative multisection computed tomography evaluation can provide necessary anatomic information in minimally invasive surgeries. This study was done to estimate the prevalence and pattern of variations of renal vasculature through contrast-enhanced computed tomography in patients referred to the radiology department of a tertiary care hospital.

**Methods:**

A descriptive cross-sectional study was conducted from 6th April 2016 to 6th April 2017. Ethical approval was taken. The triple-phase contrast-enhanced computed tomography was performed on 188 patients enrolled through convenient sampling. The images were evaluated in unenhanced, arterial, and venous phases for the vascular variants. Data were analyzed based on the anatomical types of variations and descriptive statistics such as frequency and percentage using the Statistical Package for the Social Sciences.

**Results:**

Out of the 188 patients, 60 (31.9%) had accessory renal arteries. The most common variant was hilar arteries which comprised 38 cases (20.2%) whereas polar arteries were present in 21 (11.1%) cases and the capsular artery was present in one (0.5%) case. Early bifurcation of the renal artery was noted in 15 (8%) cases with 10 (5.3%) on the right and 5 (2.7%) on the left side. Twelve (6.3%) cases of the double right renal vein were noted whereas retroaortic left renal vein was noted in only 4(2.1%) cases.

**Conclusions:**

Based on our study, almost one in three patients had accessory renal arteries and eighty-five out of a thousand patients had variants of renal veins.

## INTRODUCTION

Multi-detector Computed Tomography (MDCT) is the choice for preoperative evaluation of living renal donors Before its introduction with multiple reformations techniques like in the modern scanners, the exact anatomy of the relevant organs and their vasculature were in dilemma for surgeons. So, small and conspicuous vasculature went unnoticed, increasing the postoperative and the intraoperative complications.^1^

Multiple renal arteries occur on the left side in 26% of people and on the right side in 23%. Renal veins have a more uniform anatomic pattern than renal arteries. Ninety-two percent of people have one renal vein on each side. The left renal vein is approximately 7.5 cm and the right is 2.5 cm long.^2^ Therefore, preoperative multisection computed tomography (CT) evaluation can provide necessary anatomic information in minimally invasive surgeries.

This study was done to estimate the prevalence and pattern of variations of renal vasculature among patients referred to a radiology department.

## METHODS

This descriptive cross-sectional study was conducted among the patients referred to the Department of Radiodiagnosis, Tribhuvan University Teaching Hospital (TUTH)for a Contrast-Enhanced Computed Tomography (CECT) abdomen (triple phase). The study was done from 6th April 2016 to 6th April 2017. Ethical approval was taken from Institutional Review Committee (IRC) of TUTH (Reference Number: 325 (6-11-E). Patients with a previous history of any abdominal surgery and patient suffering from any chronic illness which affects the genitourinary system were excluded. Convenient sampling was done. The sample size was calculated using the formula,

$$\begin{array}{ll} \text{n} & \text{= }{\text{Z}}^{\text{2}}\times \text{p}\times \text{(1}-\text{p)}/{\text{e}}^{\text{2}}\\ & \text{= }{\left(1.96\right)}^{\text{2}}\times 0.5\times 0.5/{(\text{0.08)}}^{\text{2}}\\ & \text{= }150 \end{array}$$

Where,

n = required sample size
Z = 1.96 at 95% Confidence Interval
p = population proportion, 50%
e = margin of error, 8%

Taking a nonresponse rate of 20%, the sample size was calculated to be 180. However, 188 participants were taken into the study.

Informed consent was taken from all participants. The history of any other medical issues, previous surgery, hypertension, and diabetes as well as genitourinary pathologies were asked. The proforma was filled for each patient. Scans were performed in a Siemens Somatom 128 slice CT machine.

The patient was asked to drink 600 ml of plain water 30 min before and then 400 ml just before the examination. The scanning area was selected from the dome of the diaphragm to the iliac crest. The scan parameters were detector collimation: 0.6x128, pitch: 0.85, Kv: 120, mAs: 200, and recon slice and interval were 5x5mm. 80-100ml of intravenous contrast was given at the rate of 3.5 ml/s. The three-phase scans were done namely; arterial (18-20 sec), the portal (45-55sec), and venous (65-70sec) phases. The most essential step in CT angiography is the acquisition of raw data. The value and accuracy of the volumetric study will depend on this first stage.^3,4^ Exact timing of image acquisition and meticulous management of the patient including preparation, positioning, and contrast injection technique is crucial to obtain images of good quality.

Data were collected and analyzed using the Statistical Package for the Social Sciences. Descriptive statistics were used to analyse the data and the results were expressed as frequency and percentage.

## RESULTS

Of the total 188 patients undergoing MDCT, 87 (46.3%) were male and 101 (53.7%) were females. Sixty (31.9%) cases had accessory renal arteries. Hilar arteries were the most common type of accessory renal artery seen in 38 (20.2%) cases. The capsular artery was found in only one (0.5%) case (Table 1). Six (3.2%) cases had bilateral and 16 (8.5%) had unilateral polar artery out of which 11 (5.9%) had single accessory arteries and 5 (2.7%) cases had more than one accessory polar arteries. Out of 38 cases with hilar arteries; 30 (7.9%) were unilateral and 8 (21.1%) cases with bilateral hilar arteries. Single and unilateral hilar arteries were present in 28 (73.7%) cases whereas multiple accessory hilar arteries were seen in 2 (5.3%) cases.

**Table 1:** Types of accessory renal arteries.

Types of accessory renal arteries	Frequency n (%)
Polar arteries	21 (11.1)
Hilar arteries	38 (20.2)
Capsular	1 (0.005)
Total	60 (31.9)

In our study, the mean length of the left kidney was 9.94 cm (minimum of 7.4 cm and a maximum of 12 cm). The mean length of the right kidney was 10.21cm (minimum of 7.3 cm and a maximum of 12.7 cm). The mean diameter of the right renal artery was approximately 5.6 mm with a minimum diameter of 3 mm and a maximum diameter of 8.1 mm whereas the mean diameter of the left renal artery was 5.7 mm with a minimum diameter of 3.7 mm and a maximum diameter of 8.3mm.

The distance between the origin of the renal artery and its first bifurcation was approximately 36.72 mm (with a minimum of 4 mm in the right and maximum of 71.6 mm) whereas 28.94 mm in the left (minimum of 5 mm and a maximum distance of 62.8 mm). On the right side, 12 (6.3%) cases were noted to have early renal artery bifurcation meaning that the distance between the renal ostium and the first branching is less than 10mm.

Twelve (6.3%) cases of the double right renal vein were noted whereas retroaortic left renal vein was noted in only 4 (2.1%) cases (Table 2).

**Table 2:** Variations of renal veins.

Types of variations	Side	Frequency n (%)
Double renal veins	Right	12 (6.38)
Retro-aortic left renal veins	Left	4 (2.1)

The mean distance between the IVC and right renal artery bifurcation was 10.4 mm with a minimum of 3.1mm medial to IVC margin and a maximum of 44 mm from the lateral margin of IVC. The distance between the confluence of right renal veins and IVC is approximately 12.808 mm with a minimum of 3 mm and a maximum distance of 29 mm in this study. The distance between the confluence of left renal veins and the left lateral margin of the wall of the aorta was 18.6 mm with a minimum distance of 7 mm and a maximum distance of 42 mm. Only 5 (26.6%) cases were noted with a prominent gonadal vein.

## DISCUSSION

Conventional angiography is the gold standard for the detection of renal vascular variants. However, Computed Tomography Angiography (CTA) and Magnetic Resonance Angiography (MRA) is emerging as new tools for preoperative imaging. Angio-CT permits higher resolution, given that MRA pulse sequences do not allow scanning with a thickness of 1 mm or less. Some artifacts are more likely to occur in MRA and can compromise image quality, such as phase encoding artifacts, vascular pulsation, and chemical shift artifacts at fat-soft tissue interfaces, especially in the retroperitoneum. All these artifacts can cause misdiagnosis of small vessels. Besides, the cost of MRA makes it a less accessible technique in most centers.

The main target of the unenhanced phase is to locate the kidneys, rule out calculi and provide a baseline study to compare the enhancement of eventual lesions. The optimal timing for the contrast-enhanced phases depends on the volume of contrast material, the administration rate and the individuals cardiac output, these factors mandate a delay time between the start of the contrast introduction and the beginning of the scan.^5^

Once raw data are acquired, the next step is to generate axial images through a process. Axial data can be directly analysed or used to produce multiplanar images. Multiplanar reformation (MPR) is a technique that processes information from axial CT images to create non-axial two-dimensional coronal, sagittal, oblique or curved plane images. MIP (maximum intensity projection) is a reconstruction algorithm that selects and displays only the voxels with highest attenuation value of a selected slab in the visualization plane.^6^

To include long segments of vessels in CT angiography, thick-slab MIP images can be helpful, but small arteries should be evaluated with thin sections viewed in sequence because usually they are not visible because of their low attenuation. Volume rendering is an effective modality for the 3D display of imaging data. It assigns opacity values on a full spectrum from 0% (transparency) to 100% (opacity) along an artificial line of sight projection.^6–8^ Radiologists should be aware of advantages and disadvantages of these techniques and how they complement each other: MIP excels at displaying vascular maps and small intraparenchymal vessels in enhancing organs (Fig. 1), whereas volume rendering is better at depicting small extraparenchymal vasculature, grading intraluminal stenosis with calcium and demonstrating 3D relationships between structures.^8–10^

In the study performed by Rydberg et al. 71% of kidneys have one artery and 24% have two arteries.^5^ Of the two arteries, 12% contain two hilar arteries, 7% contain one hilar and one superior polar artery, 5% contain one hilar, and one inferior polar artery. Only 5% of kidneys contain three (4%) or more (1%) renal arteries.^5^ Compared to the study of Rydberg et al. more accessory arteries were seen in this study which could be due to a large number of cases or ethnic variations. The importance of mentioning polar arteries is that if inadvertently cut during the surgery can lead to excessive bleeding or renal infarction. In the study performed by Rydberg et al,^5^ double and triple veins usually are seen in the right kidney and are present in 15% of donors. The circumaortic and retroaortic veins (present in 6% and 3% of donors, respectively) are the most common major venous variants in the left kidney and are related to the embryologic development of the IVC. Some authors also prefer to take a scan in the later arterial phase to minimize the radiation dose to the patient. However, this can lead to accidentally missing the retroperitoneal veins which fill slowly during the venous phase. If missed during the late arterial phase can compromise surgery.

In a study performed by UC Turba et al.^11^ at the University of Virginia, the mean right renal artery ostial diameter were M/F= 5.06/4.59 mm, and the mean left ostial renal diameter were M/F= 5.14/4.66 mm. The maximum diameter of the accessory renal artery was 3 mm which is one of the hilar arteries and is the most common variation. There were not many variations in the diameter of the renal arteries of both sides in comparison to our study with the Ostia of bilateral renal arteries measured 5-6mm.

The major limitation of this study is that surgical confirmation for the cases could not be obtained as the cases are taken not only from the donor's kidneys. The number of cases is another limitation of the study. Continuation of data pooling from multiple institutions would be highly valuable to know the prevalence of renal vascular anomalies in our population.

## CONCLUSIONS

The renal vasculature anomalies are commonly encountered during live kidney donor workup. In this study, approximately almost one in three cases were found to have accessory renal arteries. The most common variation were hilar arteries. Few cases had variations of renal vein
